# BDNF Polymorphism: A Review of Its Diagnostic and Clinical Relevance in Neurodegenerative Disorders

**DOI:** 10.14336/AD.2017.0717

**Published:** 2018-06-01

**Authors:** Ting Shen, Yuyi You, Chitra Joseph, Mehdi Mirzaei, Alexander Klistorner, Stuart L. Graham, Vivek Gupta

**Affiliations:** ^1^Faculty of Medicine and Health Sciences, Macquarie University, Australia; ^2^Save Sight Institute, Sydney University, Sydney, Australia; ^3^Faculty of Science and Engineering, Macquarie University, Australia

**Keywords:** BDNF, polymorphism, neurodegenerative diseases, glaucoma, multiple sclerosis, Alzheimer’s disease

## Abstract

Brain-derived neurotrophic factor (BDNF) has a unique role in the neuronal development, differentiation, and survival in the developing and adult nervous system. A common single-nucleotide polymorphism in the pro-region of the human BDNF gene, resulting in a valine to methionine substitution (Val66Met), has been associated with the susceptibility, incidence, and clinical features of several neurodegenerative disorders. Much research has been dedicated to evaluating the effects of polymorphism in the past decade, and functional effects of this genetic variation. A better understanding of how this naturally occurring polymorphism associates with or influences physiology, anatomy, and cognition in both healthy and diseased adults in neurodegenerative conditions will help understand neurochemical mechanisms and definable clinical outcomes in humans. Here we review the role and relevance of the BDNF Val66Met polymorphism in neurodegenerative diseases, with particular emphasis on glaucoma, multiple sclerosis (MS), Alzheimer’s disease (AD) and Parkinson’s disease (PD). Several controversies and unresolved issues, including small effect sizes, possible ethnicity, gender, and age effects of the BDNF Val66Met are also discussed with respect to future research.

Neurotrophins are a family of growth factors crucial for the regulation of neuronal maintenance, differentiation, and survival. Among the neurotrophins, the dysregulation of brain-derived neurotrophic factor (BDNF) has been implicated in enhanced vulnerability to neurodegenerative diseases. The deficiency of BDNF, though not necessarily the initial trigger for the disease process, may result in accelerated cell damage and other associated symptoms. A naturally occurring polymorphism in the human BDNF gene leading to an amino-acid residue substitution from valine (Val) to methionine (Met) at position 66 within the pro-region of BDNF, results in a functional single-nucleotide polymorphism (SNP) that possibly results in altered BDNF activity-dependent secretion [1]. The role and association of the BDNF polymorphism with various neuropsychiatric disorders has been extensively reviewed [2]. Several studies have emerged implicating the association or otherwise of this polymorphism with neurodegenerative disorders of the central nervous system (CNS), such as Alzheimer’s disease (AD), multiple sclerosis (MS), and Parkinson’s disease (PD), and also glaucoma which is characterised by optic nerve degeneration. This review comprehensively assesses the literature on BDNF polymorphism in these pathological conditions with a view to better understand its potential involvement or association with these disorders.

## Brain-derived neurotrophic factor (BDNF) and neurodegeneration

The neurotrophin family consists of four major types of structurally related proteins with similar function, namely the nerve growth factor (NGF), brain-derived neurotrophic factor (BDNF), neurotrophin-3 (NT-3) and neurotrophin-4 (NT-4). The neurotrophins have similar molecular weights (13.2-15.9 kDa), isoelectric points (within the range of 9-10), and share approximately 50% of the identity in primary structure [3, 4]. Among these neurotrophins, BDNF has emerged as a major regulator for several types of neurons, including sensory neurons, retinal ganglion cells, spinal motor neurons, certain cholinergic neurons, and some dopaminergic neurons [5]. It has been reported that some BDNF precursor (pro-BDNF) is released extracellularly and mediates tropomyosin receptor kinase B (TrkB) receptor phosphorylation [6]. The synthesis of BDNF is influenced by neuronal activity in the brain and plays a unique role in synaptic transmission and plasticity. BDNF is widely expressed in the CNS, and its expression is decreased in several neurodegenerative diseases as demonstrated by post-mortem studies, including in AD [7-9], PD [10], and Huntington’s disease (HD) [11]. It has also been demonstrated that BDNF concentration is reduced in individuals with major depression and increases after antidepressant drug treatment [12].

The trophic effects of BDNF are mainly mediated through its high-affinity receptor TrkB, which upon activation undergoes phosphorylation and dimerization of its specific intracellular tyrosine residues. In the developing hippocampus, BDNF and TrkB signalling plays a pivotal role in mechanisms associated with neuronal plasticity such as long-term potentiation (LTP). LTP represents a persistent strengthening of synapses in between nerve cells that plays a key role in learning and memory formation [13]. While the application of exogenous BDNF was shown to facilitate LTP, the decrease of BDNF levels by gene knock-out in mice attenuated LTP in the hippocampus [14, 15]. BDNF also binds to its low-affinity receptor p75 which is a member of the tumour necrosis factor receptor (TNFR) superfamily. The p75 activation at the cytoplasmic region results in subsequent activation of NFkB which induces programmed cell death (apoptosis) [16]. The neurotrophins together with their receptors play pivotal roles in the regulation of various cell signalling pathways linked to growth, differentiation, survival, and apoptosis [17].

The exogenous administration of BDNF could be a valuable therapeutic approach for related diseases. It has been reported previously that intraventricular administration of recombinant human BDNF (rhBDNF) partially protects the basal forebrain cholinergic neurons from axotomy-induced degenerative changes in a rat model [18]. Similarly, intravitreal injection with BDNF can enhance retinal ganglion cell (RGC) survival in the cat optic nerve crush model [19]. However, while an insufficient dose might not produce the desirable effects, an indiscriminate overload of BDNF may also lead to inflammation, reduced cell survival or even potentially exacerbated seizure development [20]. Thus, future studies are warranted to explore the optimal dosage and administration methods and novel approaches to minimise non-specific side effects for potential clinical application.

## BDNF polymorphisms

The BDNF gene is localized on chromosome 11 band p13 [4] and comprises 11 exons and 9 functional promoters [21]. It can produce at least 34 mRNA transcripts responding to a variety of stimuli [22].

The typical and most widely used method for genotyping the BDNF polymorphism is by polymerase chain reaction - restriction fragment length polymorphism (PCR-RFLP). Enzymatic digestion with the specific restriction enzyme (RE) is performed on PCR products and the genotype of the subject can be identified subsequently from the gel electrophoresis results [23, 24]. PCR genotyping method of amplified refractory mutation system was reported to be cost-effective, efficient, and as reliable as PCR-RFLP for detecting BDNF polymorphisms [25]. Direct sequencing is also able to detect the genetic alteration and is commonly used to confirm the doubtful results obtained after the RE digestion [23, 26]. In addition to small scale projects aiming to identify a single polymorphism, several genotyping approaches such as TaqMan assay, fluorescence polarization, pyrosequencing, SNP gene chip based assay, and mass spectrometry based assay and so on have also enabled medium to high throughput (hundreds to tens of thousands of genotypes a day) as well as multiple SNP detection and was described extensively in previous reviews [27-29].

### Val66Met

The understanding of BDNF function in humans has greatly benefited from the identification of a SNP in the BDNF gene that causes a valine (Val) to methionine (Met) substitution at codon 66 (Val66Met, c.196G>A, dbSNP: rs6265). In this Met variant form of BDNF carriers, that is, BDNF Val/Met heterozygotes and Met/Met homozygotes, the pro-domain structure of the gene is altered. Though this alteration would not necessarily change the intrinsic biological activity of the mature BNDF protein, the polymorphism can lead to improper protein folding and a reduced binding of the mature BDNF to its receptor TrkB, causing impairments in hippocampal function [1]. The polymorphism can potentially alter BDNF protein-protein interactions, binding affinities, localisation, or conformational stability of the protein. Whether the polymorphism has any significant impact on the proteome profile or posttranslational modifications of various proteins in the neuronal tissues or body fluids is currently unknown. It has been reported that BDNF Val66Met may have roles in susceptibility for migraine, especially for migraine with aura (MA) subtype [30]. The Val66Met polymorphism is not known to occur naturally in other vertebrate species except the human so far [31].

**Table 1:** Allele and genotype distributions for the Val66Met polymorphism of Brain-derived neurotrophic factor (BDNF) in healthy subjects in different ethnicity.

Ethnicity	Cohort	Total N	Reference	Allele frequency		Genotype		
				A (Met) %	G (Val) %	A/A (Met/Met) %	G/A (Val/Met) %	G/G (Val/Val) %
Asian	Korea	244	Pivac et al. 2009	46.3	53.7	23.4	45.9	30.7
	Japan	657	Fukumoto et al. 2009	39.0	61.0	15.0	47.0	38.0
	Japan	275	Nishimura et al. 2009	42.5	57.5	17.1	50.9	32.0
	China	239	Bian et al. 2005	45.4	54.6	21.3	48.1	30.5
	Japan	471	Matsushita et al. 2005	44.5	55.5	20.8	47.4	31.9
	Japan	151	Itoh et al. 2003	41.1	58.9	15.9	50.3	33.8
Caucasian	Romania	1124	Vulturar et al. 2016	19.3	80.7	4.0	30.5	65.5
	Poland	193	Nowak et al. 2014	15.0	85.0	1.0	30.0	69.0
	Croatia	556	Pivac et al. 2009	19.5	80.5	3.4	32.4	64.2
	Italy	233	Guerini et al. 2009	20.6	79.4	4.3	32.6	63.1
	Italy	37	Liguori et al. 2009	17.6	82.4	2.7	29.7	67.6
	USA	250	Zhang et al. 2006	18.8	81.2	4.0	29.6	66.4
	Finland	920	Vepsalainen et al. 2005	13.0	87.0	2.0	24.0	75.0
	USA	194	Bodner et al. 2005	19.0	81.0	3.0	32.2	64.7
	USA	392	Parsian et al. 2004	28.0	72.0	2.0	53.0	45.0
	Spain	218	Combarros et al. 2004	19.0	81.0	3.7	30.7	65.6
	USA	133	Egan et al. 2003	18.0	82.0	4.5	27.1	68.4
	Italy	111	Ventriglia et al. 2001	29.7	70.3	8.1	43.2	48.7

Several studies have described the distribution of Met allele carriers in healthy participants across the world. The Val66Met polymorphism frequency varies depending on both region and ethnicity. A 0 to 72% Met allele frequency was presented in a population genetic study of BDNF [32]. Specifically, the Met carriers make up approximately 30% to 50% of Caucasian populations in cohorts from the U.S. (31.6%), Croatia (35.8%) and Italy (51.30%) [33-35]. Whereas among Asian populations, almost 70% of people in China (69.5%), Japan (66.20%) and Korea (69.30%) carry the Met variant form of BDNF gene [24, 33, 36] (See Table. 1). Significant ethnic differences for the Val66Met were also reported in these studies, providing a possible explanation for the various conflicting associations demonstrated between the polymorphism and neuronal disorders.

### Other BDNF gene variants

Besides Val66Met, other BDNF gene polymorphisms are also reported to exhibit possible positive or negative association with various deficits indifferent neuronal disorders. The C270T (rs56164415) polymorphism located in the non-coding region of BDNF was found to be associated with increased risk of late-onset Alzheimer’s disease (LOAD) and also linked with susceptibility to and the onset of MS [37, 38]. The rs2030324 polymorphism was specifically shown to affect visual cognitive processing in MS patients [39]. However, other studies suggested no significant associations between these polymorphisms and risks for neurological diseases [34, 40-44].

## The effects of Val66Met polymorphism on neurodegenerative disorders

Since neurotrophins are essential for neuronal function, development, survival and regeneration, gene variations encoding these proteins can confer susceptibility to neurodegenerative disorders [45].

**Figure 1.**
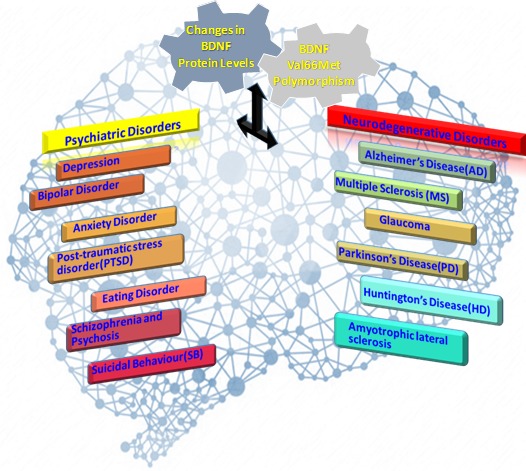
Changes in Brain-derived neurotrophic factor (BDNF) protein levels and BDNF Val66Met polymorphism as modifiers of neurodegenerative disorders and psychiatric disorders.

The Met allele was hypothesized to impair intracellular trafficking and synaptic localization of mature BDNF and significantly weaken the activity-dependent secretion of BDNF (up to 30%) while keeping the constitutive secretion of BDNF unaffected [1]. A similar reduction of activity-dependent BDNF secretion was also observed in neurons derived from BDNF Val66Met knock-in mice [46]. However, inconsistent results of higher [47, 48], lower [49] as well as similar [50, 51] levels of BDNF serum concentration in Met carriers have been reported in genetic association studies. The conflicting results could be due to psychopathology, ethnicity and/or gender difference [52].

Magnetic resonance imaging (MRI) analysis indicated that the BDNF polymorphism has an impact on the anatomy of the hippocampus and prefrontal cortex in healthy human subjects. The Met carriers exhibited significant bilateral decrease of hippocampal grey matter volumes compared to Val homozygotes, suggesting a modifying effect of the functional variation in brain morphology related to learning and memory [53]. Interestingly, a longitudinal study illustrated that although the BDNF Val homozygotes outperformed the Met carriers at the first testing session for cognitive function, they showed a substantial decrease in performance after 10-years’ time at the second session [54]. This result is partially consistent with an earlier study demonstrating a better performance in Met homozygotes in older age [55], suggesting that the BDNF impact on cognitive function may alter during the human lifespan and that the Met allele may provide neuronal protection during later stages of life. A recent study also reported a positive effect of Met homozygosity on regional grey matter volume (rGMV) in healthy Japanese children and adolescents, suggesting a possible role the Val66Met have on human development [56]. These emerging results continue to substantiate the role of BDNF Val66Met in the susceptibility and progression of neurodegenerative diseases.

### Glaucoma

Glaucoma is a group of complex neurodegenerative diseases characterised by progressive degeneration of retinal ganglion cells (RGCs), cupping of the optic nerve head (ONH) [57], and abnormal thinning of retinal nerve fibre layer (RNFL) [58], resulting in irreversible loss of vision and visual sensitivity. It has been estimated that almost 80 million people will suffer from glaucoma by 2020 with approximately 14% being bilaterally blind, making glaucoma the second leading cause of irreversible blindness worldwide [59].

A strong genetic basis of the disease predisposition is supported by several lines of studies in both primary open angle (POAG) and closed angle glaucoma [60-63]. However, these susceptibility genes that have been identified so far account for just a small proportion of glaucoma cases and have only modest effect size in elucidating the risk for glaucoma. There is an opportunity to explore disease modifiers and cross-talk between already identified gene variants for a better understanding of the disorder.

Accumulating evidence suggests that molecules with neurotrophic actions such as BDNF affect RGC development and maintenance. Inside the retina, BDNF is expressed by cells in the inner nuclear layer (INL) and ganglion cell layer (GCL) [64]; its cellular target TrkB receptor is produced by RGCs and amacrine cells [65] that support the BDNF trophic actions. The axonal transport of BDNF and TrkB receptor are suggested to be obstructed in both acute and chronic experimental glaucoma models, suggesting that deprivation of BDNF to RGCs contributes to the pathogenesis of neuronal loss in glaucoma [66].

Several studies have shown that BDNF acts as a potent neuroprotectant for RGCs after optic nerve (ON) injury [19, 67] and after increased IOP which mimics the primary open-angle glaucoma (POAG) [68, 69]. In most studies, BDNF was administered to the intraocular tissues by intravitreal injection. However, a recent study utilizing topical eye application of BDNF eye-drops on mice showed a similar protective result in animals with experimental glaucoma [70]. In addition, overexpressing the BDNF gene by introducing the modified adeno-associated viral (AAV) vector into the rat retina also demonstrated a similar neuroprotective effect for laser-induced experimental glaucoma, providing support for the concept of potential gene therapy applications in human glaucoma [71].

As a functional genetic alteration for BDNF, the Val66Met polymorphism was reported to be significantly associated with progression of POAG determined by the nerve fibre layer analyser (GDx) but not with the risk of POAG in a Polish cohort [72]. This result suggested that Val66Met may have a role in glaucomatous RGC loss.

### Multiple sclerosis (MS)

Multiple sclerosis (MS) is a chronic autoimmune, inflammatory neurological disorder of the CNS. MS progression leads to demyelination, axonal degeneration and subsequent neurological impairment and disability [73]. More than 2.3 million people worldwide were affected with MS by 2013, and up to 60% of patients will need walking assistance within 20 years after the onset of disease [74]. The disease typically presents in young adults with an average age of onset of 29 years and affects twice as many women as men [75]. Several ancillary tests can be used to support clinical diagnoses, such as brain MRI, sensory evoked potential testing, cerebrospinal fluid analysis and serologic testing [73].

Since genetic factors were demonstrated to affect the susceptibility to MS, series of genome-wide association studies have been performed and multiple SNPs that are associated with MS have been identified, including polymorphisms in genes for interleukin 2 receptor alpha, interleukin 7 receptor, rs703842 and rs10876994 on chromosome 12, and CD40 on chromosome 20 [76, 77]. However, these SNPs individually exert only modest effects on the overall MS risk and were reported to have no association with the MS disease severity measures [78].

A two-year longitudinal study showed higher peripheral BDNF mRNA levels in relapsing-remitting multiple sclerosis (RRMS) patients with Met alleles with respect to Met carriers of healthy controls, suggesting that the BDNF polymorphism may affect the peripheral BDNF production during RRMS [79]. Another study also reported opposite effects on BDNF polymorphism association with parieto-prefrontal network activation and hippocampal disengagement in controls and RRMS subjects by working memory challenges [23].

The attempts to explore the influence of BDNF Val66Met polymorphism on the brain of MS patients have produced conflicting results. In an earlier study, RRMS patients with Met alleles, relative to healthy controls with matching age, gender, and educational levels or MS patients with two Val alleles, exhibited a lower volume of cerebral grey matter (GM). The Met carriers also had a higher risk to develop global grey matter atrophy than the Val/Val homozygous RRMS patients [80]. On the contrary, several later studies showed an opposite result where the Met carriers had increased grey matter volume in the brain and had possible protective effect for grey matter preservation in MS [81, 82]. The discrepancies might result from the differences in cohort selection as the former was carried out in RRMS patients without treatment and with low disability while the latter studies had cohorts of treated MS patients. It has also been suggested that differential effects could have been caused by Met allele on subsequent brain volume change, by directly affecting neuronal BDNF secretion, and indirectly influencing the inflammatory activity or possibly affecting secondary secretion of BDNF by immune cells, although the effect of oedema on lower brain atrophy could not be excluded [83]. Further, an fMRI study showed that the Val66Met polymorphism has differential effects on the hippocampal memory system of healthy controls and RRMS patients. Particularly, in RRMS patients, the Met allele carriers showed increased hippocampus posterior cingulate cortex connectivity in comparison with Val homozygotes during retrieval phase of the episodic memory task while the reverse was true for controls [84].

In a Polish cohort, the Val homozygotes were found to have increased MS susceptibility in females and earlier onset of disease in males, suggesting a gender difference in the effect of the polymorphism on MS [38]. The BDNF Val66Met polymorphism showed no significant influence on the severity of depression in MS patients, though the result was in disagreement with the findings based in the general population [85]. Negative results were also reported in MS susceptibility and clinical course in human cohorts from the UK, Norway, and Spain [44, 86, 87]. Future studies with larger sample sizes are needed, particularly with a special focus on gender and ethnic differences.

Many epidemiological studies have reported a higher prevalence and incidence of MS in Caucasian populations than in Asian [88, 89]. However, the prevalence of neuromyelitis optica (NMO), a more severe demyelinating disorder, is higher in Asian population [90-92]. Since the BDNF Met allele frequency is substantially higher in Asians compared to Caucasians, one would expect this genetic variation might be associated with a higher prevalence if it was associated with the pathogenesis of MS. Larger studies of different ethnic groups with matching age, sex ratio, MS subtypes, etc. and with high statistical power need to be conducted to test the hypothesis.

## Alzheimer disease (AD)

Alzheimer disease (AD) is an irreversible neurodegenerative disorder associated with specific pathological changes leading to neurodegeneration and progressive symptoms of dementia. AD is clinically characterized by memory impairment, loss of cognitive functions and behavioural impairment, and pathologically by the presence of beta amyloid (Aβ) andhyperphosphorylated Tau protein plaques along with neurofibrillary tangles in the brain [93]. It has been estimated that 26.6 million people worldwide were afflicted with AD in 2006, and the global prevalence is expected to quadruple to approximately 106 million by the year of 2050 [94]. Despite major efforts in research and drug development, no effective treatments or drugs have been developed to prevent AD progression [95].

Attempts are in progress to flag and diagnose individuals with high-risk for AD in an early stage by genetic screening tests, and such risk-assessment profile would enable future personalized treatment plans. Several gene missense mutations predisposing to increased AD susceptibility have been identified and validated. For instance, genes encoding amyloid precursor protein [96], presenilin 1 [97], and presenilin 2 [98] confer susceptibility to familial AD. The genetic alterations in presenilin genes are the most common cause of familial AD while the mutations in APP account for only very rare cases [99]. The ε4 allele of the apolipoprotein E (APOE) is a major genetic predisposing factor for both familial and sporadic AD [98]. However, AD is a genetically complex disorder and the overall susceptibility cannot be fully explained by the aforementioned genes. Thus, additional gene alterations and linkages may dictate the development and progression of various subtypes of AD.

Some studies suggest that there is altered function of the BDNF gene in the pathogenesis of AD. A sexually dimorphic effect of the BDNF Val66Met mutation on AD development was observed in a large sampled meta-analysis study (4,711 patients and 4,537 controls included). The female Met allele carriers depicted a higher prevalence of AD, but such an association was not found in male carriers [41]. The female BDNF Val carrier patients were also found to have delayed age of onset for the disease as well as a protective effect in regard to AD development in a Chinese cohort [24].

A case-control study in a Japanese population reported a higher Met allele frequency in AD subjects in comparison with the cognitively normal controls, suggesting that BDNF Met allele may play some role in the development of the disease [42]. In addition, it has been suggested that the BDNF Val66Met polymorphism moderates the memory decline and hippocampal atrophy in prodromal AD subjects with high baseline levels of Aβ [100]. Comparing to Val/Val homozygotes, significantly higher rates of hippocampal atrophy and accelerated episodic memory decline were observed in BDNF Met carriers with amnestic mild cognitive impairment (aMCI) and high Aβ density. However, the rate of Aβ accumulation was not moderated by the BDNF polymorphism, suggesting that the polymorphism may accelerate neurodegeneration and memory decline by influencing downstream processes, such as tau aggregation [100]. Similarly, a significant decline of hippocampal volume and memory was observed in healthy BDNF Met carriers with high Aβ levels [101].

A recent study exploring the effects of BDNF Val66Met on autosomal dominant Alzheimer’s disease (ADAD) showed greater deleterious effects of Aβ on episodic memory, hippocampal function, and tau in preclinical ADAD mutation carriers with Met alleles [102]. In addition, an early study showed that the number of individuals who were homozygous for Val allele was significantly higher in AD subjects compared to the controls, suggesting an increased risk of AD in people with two Val alleles [35].

In contrast, no association between BDNF Val66Met polymorphism and risk of LOAD was observed in Japanese [103], Chinese [104], Spanish [105], Finnish [42], Italian [106], and American [40] cohorts. The reduced level of BDNF protein in temporal neocortex region of the AD subject brains did not correlate with BDNF Val66Met [107]. These conflicting results have led to suggestions that BDNF Val66Met polymorphism may only act as a modifier in early-onset cases rather than the LOAD. However, it is to be acknowledged that the studies showing a negative result had medium to small sample size ranging from 163 to 256 AD patients, and the lack of sufficient statistical power could also lead to ambiguous outcomes. Furthermore, recent studies have revealed an association between another BDNF polymorphism C270T (where a C to T substitution occurred within the non-coding region of BDNF gene) and LOAD [37, 103, 108] raising the possibility that Val66Met may be in linkage disequilibrium with other variants either within or close to the BDNF gene.

## Parkinson’s disease (PD)

Parkinson’s disease is a progressive neurodegenerative disease featured by the death of dopaminergic neurons in the substantia nigra and the presence of Lewy bodies [109]. Like other neurodegenerative disorders showing non-mendelian inheritance, the pathogenesis of PD remains unclear, however several studies implicate a complex interplay between genetic and environmental impacts in the causation of PD [110].

A significantly reduced BDNF mRNA expression was reported in PD substantia nigra pars compacta (SNpc) compared to control neurons in an early post-mortem human study, although this reduction could partially be due to the loss of SNpc dopaminergic neurons which specifically express BDNF. Surviving dopaminergic neurons in the SNpc of PD patients express less BDNF than healthy controls, suggesting that the decrease of BDNF might contribute to the loss of nigral dopaminergic neurons and pathogenesis of PD [111]. The serum BDNF levels were also found to be decreased in PD subjects [112].

Emerging evidence increasingly indicates that the BDNF polymorphism possibly modulates the prevalence or clinical course of PD. Association studies of 20 candidate gene SNPs for PD have revealed that the Met/Met genotype frequency is higher in PD patients than healthy controls in a Japanese cohort [113]. A later onset age of 5.3 years has been reported in BDNF Met homozygotes with familial PD compared to the heterozygotes and Val homozygotes in a UK study [114]. Two more studies elucidated that the BDNF Met allele carriage is associated with a higher prevalence of cognitive impairment in PD patients, indicating that the polymorphism may be a risk factor for cognitive dysfunction in PD [115, 116]. The PD patients with Met alleles were reported to develop levodopa induced dyskinesia (LID) earlier during treatment with dopaminergic agents [117] than those with two Val alleles. There is also evidence that the BDNF genotypes independently influence the planning task performance of the tower of London (TOL) test in PD patients, even after adjustment for other possible confounding factors, however, the better performance in Met carriers was reported to be significant only in female but not in the male participants [109]. The BDNF Met carriers with PD were also demonstrated to have significantly smaller decline in set shifting (cognitive flexibility that includes the ability to shift attention between tasks) during a two-years later follow-up test compared to the Val homozygotes, indicating a possible beneficial effect of the Met allele on mental flexibility [118].

Nevertheless, conflicting results still persist as previous studies from Finland [119], Sweden [120], Taiwan [121, 122], Greece [123, 124], Poland [125], and Spain [126] have reported no association between Val66Met and the risk or clinical course of PD. PD is a complex neurological disease with a multifactorial aetiology, thus, a minor to moderate role of Val66Met in specific cases in collaboration with other genetic or environmental factors cannot be excluded. The association between BDNF polymorphism and PD remains an unresolved issue, and more rigorous population-based association studies are required.

## Conclusions and prospects

Neurodegenerative disorders bring about major socioeconomic burden for patients and society. Therefore, it is of great interest to understand the cellular and molecular pathological basis responsible, as there are currently no effective available therapeutic strategies to reverse or halt the degenerative process. In this review article, we discuss the potential involvement of BDNF polymorphism in several neurodegenerative disorders, and conclude that the evidence is mixed in terms of implicating the SNP in several common diseases. To answer the question, prospective cohort studies, especially with longitudinal assessments of the progression of neurodegenerative disorders, are needed. Its role in glaucoma has not been studied in depth, and since BDNF is critical to ganglion cell survival, there is the potential that the SNP may be a factor in certain subtypes of glaucoma.

The role of gender and ethnicity with respects to the BDNF Val66Met polymorphism and neurodegenerative disorders susceptibility can be further explored as differential allelic frequencies and sex effects of the polymorphism have been reported in various studies [36, 41]. Most of the published studies compared BDNF Val homozygotes with BDNF Met allele carriers due to the infrequency of BDNF Met homozygotes in Caucasian population cohorts [32]. Gene dosage studies, with sufficient power will compare all three genotypes (Val/Val, Val/Met, and Met/Met) and may potentially reveal whether any specific phenotypic effects in neurodegenerative disease pathology are truly associated with the BDNF polymorphism. The higher Met allele frequency and occurrence of Met homozygotes in Asian population may provide an opportunity for such studies [24, 32]. Parallel studies in BDNF knock in (Val66Met) mouse models overlaid with disease models using pharmacological or genetic approaches will help understand the role of BDNF variants in these neurodegenerative conditions and analyse the human gene-disease association studies. Animal studies will provide insights into the gene-protein and gene-phenotype correlation in a tissue specific manner.

It is also necessary to investigate how the BDNF polymorphism affects the neurons and CNS phenotypes including in the retina in normal healthy subjects and potential effects with age. BDNF polymorphism has indeed been suggested to have a role in geriatric depression [127], LOAD [1], age-related changes in reasoning skills and executive function [54, 55], goal-directed behaviour in the elderly [128], and reduced Stroop interference [129], and altered cognitive abilities in the elderly [130]. Thus, aging exacerbates susceptibility to neurological diseases and may amplify the effects of the polymorphism. It has been reported that BDNF Met allele carriers were more susceptible to AD in early adult life, while in contrast, Val homozygotes demonstrated susceptibility to AD in late life [131]. Identifying whether the Val66Met regulates long-term neurodegeneration in an age-dependent manner is therefore a crucial question to be further resolved, and could help develop potential therapeutic strategies in vulnerable groups.

In addition, the BDNF polymorphism is also associated with the physiology and morphology of several brain regions in healthy individuals. An 11% volumetric decrease of the hippocampal region (a significant reduction that is comparable to that associated with diseased conditions such as major depression [132]) has been associated with the BDNF Met allele carriage in healthy subjects [133]. It has been reported that in healthy individuals a combination of dopamine D3 receptor (DRD3) Ser/Ser genotype and BDNF Met-containing genotypes is associated with shorter interthalamic adhesion, suggesting an effect of gene-gene interaction between the two genetic variants on brain morphology in midline and medial temporal lobe structure [134]. Moreover, an increase in cortical surface area as well as related resting-state functional connectivity between the anterior insular and the dorsolateral prefrontal cortices (DLPFC) with respect to the dosage of Met allele is reported in a cohort of healthy Chinese subjects [135]. These studies in healthy volunteers may specify vulnerability factors for the development of disease processes associated with the dysfunction of particular regions in the brain. Therefore, BDNF polymorphisms carriage may potentially act as prognostic or diagnostic markers and may assist with the development of strategies for patient stratification, early diagnosis, and early evaluation of therapeutic efficiency of new medications.
